# Effect of guanidinoacetic acid on the growth performance, myofiber, and adenine nucleotide of meat-type rabbits

**DOI:** 10.5713/ab.23.0110

**Published:** 2023-08-16

**Authors:** Yuanxiao Li, Caicai Feng, Ning Liu, Jianping Wang

**Affiliations:** 1 Department of Animal Science, Henan University of Science and Technology, Luoyang 471000, Henan, China; 2 Luoyang Xintai Agro-pastoral Technology Co., Ltd, Luoyang 471400, China

**Keywords:** Adenine Nucleotide, Growth, Guanidinoacetic Acid, Myofiber, Rabbit

## Abstract

**Objective:**

This study aimed to investigate the effect of dietary guanidinoacetic acid (GAA) on the growth performance, slaughter traits, myofiber, and adenine nucleotide of meat-type rabbits.

**Methods:**

Experimental treatments consisted of control (CON) and GAA addition at 0.04% (T1), 0.08% (T2), and 0.12% (T3) of diet. A total of 240 weaned rabbits (meat-type male Chinese black rabbits) were randomly distributed into four groups with six replicates of ten rabbits each.

**Results:**

Results showed that the three doses of GAA increased (p<0.05) final body weight, carcass weight, the density and area of quadriceps femoris fiber; and T3 showed significant effects (p<0.05) on weight gain, feed/gain, and dressing percentage, and the traits of longissimus fiber, compared to CON. Dietary GAA increased (p<0.05) the meat color a* and b* in longissimus and quadriceps; and T3 showed the lowest (p<0.05) shear force of longissimus. Furthermore, GAA increased (p<0.05) the contents of adenosine triphosphate and total adenine nucleotide in longissimus and quadriceps. In longissimus adenosine triphosphate, total adenine nucleotide, and adenylate energy charges, T3 treatment was most effective (p<0.05); while T2 and T3 treatment was more effective (p<0.05) than T1 in quadriceps. Additionally, linear or quadratic responses (p<0.05) to the increased doses of GAA were found on body weight gain, meat color, total adenine nucleotide, and adenylate energy charges.

**Conclusion:**

It is concluded that GAA can be used in the rabbit diet to improve growth and carcass traits, and these are related to the high levels of muscle adenine nucleotide.

## INTRODUCTION

With global food demand increasing and feedstuff shortage, improving the growth performance and meat quality of farm animals is an unceasing topic in husbandry production. Protein composition and fat distribution in the carcass are mainly related to energy metabolism. Guanidinoacetic acid (GAA), also known as glycocyamine or guanidinoacetate, is an essential compound involved in cellular energy metabolism [1]. It is a precursor of creatine in the body and can also be used as a supplement to enhance athletic performance [2]. The contents of GAA vary in plant-based products at ~1 μg/kg and meat-based products at ~50 mg/kg [3]. Therefore, the diet containing plant-sourced ingredients may cause an inadequate intake of GAA.

Several studies have recently explored the energy regulation function of GAA in farm animals. The GAA at 1.0 and 1.5 g/kg of the diet increased egg production, carcass yield, and muscular essential amino acids in aged laying hens [4]. Also, in a low-energy diet, GAA did not compromise broiler growth [5]. Controversially, GAA had no effect on nitrogen retention in growing steers [6]; but reduced drip loss and flavor amino acids in the pork [7]. Energy metabolism is involved in adenosine triphosphate (ATP) turnover. GAA can outcompete traditional bioenergetics agents in maintaining ATP status in broilers under pre-slaughter transport stress [8]. The change in the muscular energy by GAA may further influence myofibers, but studies are very limited and showed that GAA lowered myofiber area, diameter, and density in pigs [9,10].

Farm rabbits are an important supplement for edible meat. Literature about GAA in rabbits is unavailable. The present study aimed to elucidate the effect of GAA on the growth performance, carcass yield, myofiber traits, meat quality, and ATP turnover of meat-type rabbits.

## MATERIALS AND METHODS

### Animal ethics approval

Research on animals was conducted according to the committee on animal use at Henan University of Science and Technology (No. 2021025).

### Guanidinoacetic acid and diet formulation

Commercial GAA was purchased from Beijing Gendone Biotechnology Co., Ltd. (Beijing, China) with a purity of 98%. The addition doses of GAA were 0%, 0.04%, 0.08%, and 0.12% of diet in the control (CON) and GAA treatments 1 (T1), 2 (T2), and 3 (T3), respectively. The basal diet was formulated according to the Nutrient Requirement of Meat-type Rabbits [11], and its ingredients and chemical compositions are listed in Table 1. Rabbits were fed and managed according to The Technical Specification for Feeding and Management of Meat-type Rabbits [12].

### Feeding trial and growth performance

A total of 240 Chinese black rabbits (male) at 50±1 days old with statistically nonsignificant body weight differences were randomly assigned to 4 groups with 6 replicates of 10 rabbits each. Rabbits in individual cages were fed twice a day with free access to diets and water. The feeding trial lasted for 45 d after an adjustment period of 5 d. Feed and rabbits in each replicate were weighed weekly. Feed intake, body weight gain, and feed/gain were adjusted when mortality occurred [13]). The general health of rabbits was monitored twice a day during the feeding trial. Feed intake, body weight gain, feed/gain ratio, survival rate, and diarrhea rate per replicate were calculated throughout the feeding trial according to the formulas: average daily body weight gain (g/d) = (final weight – initial weight)/(days on test × rabbits on test), average daily feed intake (g/d) = total feed intake/(days on test × rabbits on test), survival rate (%) = survival rabbits/rabbits on test)×100, and diarrhea rate (%) = (rabbits with diarrhea/rabbits on test)×100.

### Slaughter and carcass determination

At the last day of the feeding trial, 5 rabbits per replicate were randomly selected and euthanatized by injecting air into the ear vein (50 mL/rabbit). The pre-slaughter weight was measured after fasting for 8 h. Hot carcass weight was the weight of the carcass between 15 and 30 minutes after slaughter, which excluded blood, skin, distal parts of the tail, front feet, hind feet, gastrointestinal tract, and urogenital tract; but included liver, kidneys, head, lungs, esophagus, trachea, thymus, and heart. Commercial carcass weight was the weight of carcass weight for 24 hours after slaughter, and the carcass was hung in the room at 0°C to 4°C with normal ventilation. The dressing percentage was the ratio between hot carcass weight and live weight ×100. Reference carcass weight was the carcass containing only fat, meat, and bone tissues. It is the commercial carcass without liver, kidneys, and the set of organs of neck and chest.

### Muscle fiber and meat quality determination

One hour after slaughter, approximately 20 g of tissue samples was collected from the same anatomic site of quadriceps femoris and longissimus dorsi and put into 4% formaldehyde solution; then, dehydrated, buried into wax, sliced (7 mm thick), stained in Ein-hematoxylin, and sealed; observed under 40× microscopic field and photographed (Scopeimage 9.0; BIO-IMAG Co., Vaughan, ON, Canada) for determining the diameter (mm), area (10^−3^ mm^2^/fiber), and density (fiber/mm^2^) of muscle fiber. One hour after slaughter, meat color including lightness (L*), redness (a*), and yellow (b*) was measured by inserting a colorimeter (NR20XE; 3NH Co., Shenzhen, China) into the left quadriceps femoris and longissimus dorsi at the level of the 4th lumbar vertebra. The pH value of meat was obtained using a pH meter (Testo205; TESTO Co., Lenzkirch, Black Forest, Germany), 24 hours after slaughter.

Meat pieces (rectangular cross-section of 1×1 cm^2^ and 2 cm along the fiber axis) from the quadriceps femoris and longissimus dorsi were prepared and cut perpendicular to the muscle fiber by a Texture Analyser (TA.TX2; Stable Micro Systems, Surrey, UK), and the resistance value of the blade was defined as the shear force. For cooked meat rate, one hour after slaughter, the right quadriceps femoris (approximately 100 g) and whole longissimus were dissected without membrane and attached fat, weighed (raw meat weight), and cooked in boiling water for 30 min; then, the meat was dried in the air for 30 min, and weighed (cooked meat weight); cooked meat rate (%) = (cooked meat weight/raw meat weight)×100. All measurements were performed in triplicate.

### Muscle adenine nucleotide determination

The concentrations of ATP, adenosine diphosphate (ADP), and adenosine monophosphate (AMP) in the quadriceps femoris and longissimus dorsi were determined using high pressure liquid chromatography (S6000Plus; Acchrom Tech Co., Beijing, China) according to a previous study [15]. Briefly, muscle samples (approximately 150 mg) and perchloric acid (1 mL, 1.5 M) were homogenized and centrifuged at 3,000 g for 10 min at 0°C to 4°C. Then, the muscle supernatant (1 mL) was mixed with potassium carbonate (0.4 mL, 2 M) and centrifuged at 3,000 g for 10 min at 4°C. The chromatographic system consisted of a chromatographic column (Waters XBridge C18; 5 μm, 4.6 mm×250 mm), detection wavelength (260 nm), the pump flow rate (1.0 mL/min), and the column temperature at 35°C. Total adenine nucleotide (TAN) and adenylate energy charges (AEC) were calculated according to the following equations: TAN = ATP+ADP+AMP, AEC = (ATP+0.5 ADP)/TAN.

### Statistical analysis

Values were expressed as means and SEM using one-way analysis of variance of SPSS 23.0 software (SPSS Inc., Chicago, IL, USA). Differences among the means of treatments were separated by Post-Hoc multiple comparisons and Tukey’s-b test at p<0.05. The responses of determined parameters to GAA doses in T1, T2, and T3 were analyzed using linear and quadratic contrasts. A statistical unit for growth performance was from all rabbits, whereas statistical units for energy turnover, meat quality, and carcass traits were the average values of 5 slaughtered rabbits per replicate.

## RESULTS

### Growth performance

In contrast to CON, T1, T2, and T3 increased (p<0.05) final body weight gain and T3 showed a more pronounced (p< 0.05) effect than T1 (Table 2). T3 had a better (p<0.05) effect on weight gain than CON and T1. Also, T3 had the lowest (p<0.05) feed/gain among treatments. No statistical differences were found in the survival rate and diarrhea of rabbits. There was a linear (p = 0.022) increase in body weight gain with the increased GAA doses.

### Carcass and muscle fiber traits

The GAA addition in T1, T2, and T3 increased (p<0.05) hot carcass weight and commercial carcass weight, compared to CON (Table 3). T3 had greater (p<0.05) hot carcass weight than T1, but for commercial carcass weight, there were increments (p<0.05) with the doses of GAA. T3 had the greatest (p<0.05) dressing percentage among all groups. T3 increased (p<0.05) the fiber density of longissimus dorsi than CON. T2 and T3 increased (p<0.05) longissimus dorsi area than CON. Similarly, the fiber density and area of quadriceps femoris were increased (p<0.05) in GAA groups. T3 showed more pronounced (p<0.05) effects on the fiber area of longissimus dorsi and quadriceps femoris than other GAA groups. Additionally, with the increased GAA doses, there were linear (p≤0.048) increases in pre-slaughter weight, commercial carcass weight, the density and area of longissimus dorsi fiber, and the area of quadriceps femoris fiber.

### Meat quality

In contrast to CON, meat color L* and a* in longissimus dorsi were increased (p<0.05) in GAA groups (Table 4), while pH and shear force were decreased (p<0.05). Among the GAA groups, T2 and T3 had a more pronounced (p<0.05) effect on L* than T1; and T3 showed the lowest pH (p<0.05). For quadriceps femoris, only meat color a* was increased (p< 0.05) in GAA groups, compared to CON; and T3 showed a more pronounced (p<0.05) effect than other GAA groups. There was a quadratic (p = 0.042) relationship between the meat color L* of longissimus dorsi and GAA doses, and a linear (p = 0.035) response on the a* value of quadriceps femoris.

### Muscle adenine nucleotide

The dietary GAA increased (p<0.05) the concentrations of ATP, ADP, TAN, and AEC in longissimus dorsi (Table 5), compared to CON; and T3 showed more pronounced (p< 0.05) effects on these parameters than T1 and T2. Similarly, in contrast to CON, increased (p<0.05) effects were found on the concentrations of adenine nucleotide in quadriceps femoris; and T3 had greater (p<0.05) effects on ATP, TAN, and AEC than T1. Linear (p≤0.025) responses to the increased GAA doses were found on ATP, ADP, and TAN in longissimus dorsi; a linear (p = 0.020) effect on AMP and a quadratic (p = 0.042) effect were found on AEC in quadriceps femoris.

## DISCUSSION

In the body, GAA is methylated as creatine to ensure a sufficient supply of high-energy molecules to various demanding cells, especially myocytes. In fast-growing animals, however, it is estimated that the synthesis of the body only covers around two-thirds of the daily creatine required [8,16]; coupled with the negligible contents in plant-based diets, the remainder of GAA must be supplied by adding to the feed [17]. Indeed, this is supported by the present study and some literature. In the present study, GAA added at 0.04%, 0.08%, and 0.12% improved weight gain and feed efficiency of growing rabbits. Literature about the effect of GGA on rabbit growth is unavailable. In broilers, GAA addition at 0.067% with sparing arginine at 150% restored the feed intake, weight gain, and gain/feed [5]. Also, GAA at 0.10% or 0.15% increased egg production, serum creatine level, and brain dopamine, but decreased brain gamma-aminobutyric acid of aged laying hens [4]. In growing/finishing pigs, GAA added from 0.03% to 0.3% increased weight gain and feed intake, and the high dose reached optimum gain/feed [9,18]. Paradoxically, GAA had no significant effect on growth performance or nitrogen retention in steers, gilts, and broilers [6,19,20]. Additionally, the dietary GAA in the present study non-significantly decreased diarrhea rate; whether GAA influences the intestinal barrier deserves further study.

The increased body weight gain in GAA groups of the present study consequently affected the carcass weight and dressing percentages. In chickens, GAA increased the carcass yield, and the essential amino acids in the breast and thigh muscles [4,21]. In pigs, literature about the effect of dietary GAA on carcass traits is inconsistent, reflecting on the increases in the muscle weight and loin area of longissimus dorsi [9,22], the decreases in mandibular fat index and back-fat thickness area [10], and no effects on carcass weight, carcass length, and lean percentage [18]. The changes in muscle yield in GAA treatments may ascribe that abundant energy supply generated from the cascades of GAA to creatine, phosphocreatine, and ATP.

Importantly, in the present study, the addition of GAA increased the muscle fiber density and area of longissimus dorsi and quadriceps femoris. As known, the main editable part is the skeletal muscle which accounts for proximately 40% of the carcass [23]. The improvement of skeletal muscle and its quality is the focus of meat-type animal production. The increases in the number and volume of myofiber in the present study indicate that GAA can improve muscle development. However, Zhu et al [10] reported that GAA lowered myofiber cross-sectional area and fiber diameter in longissimus dorsi muscle, but upregulated the mRNA expression of myosin heavy chain I in finishing gilts. Also, Lu et al [9] found that GAA lowered cross-sectional area and fiber density; upregulated the expression of myosin heavy chain gene, myogenic determination, and myogenic factor 5 in longissimus dorsi; but downregulated the expression of myostatin in longissimus dorsi and fatty acid synthase in the liver of pigs. It is curious why GAA increased muscle yield, but without consistency effects on fiber area and density as mentioned in the literature, which deserves more studies.

Furthermore, in the present study, the meat quality including shear force, pH, and meat color was positively affected by the dietary GAA. Similar results were found in other animals. In broilers, GAA increased the moisture and creatine concentration in the breast meat [5]. In pigs, GAA improved water holding capacity; and lowered shear force, drip loss, b* value, and free amino acid concentration [7,9,10]. In lambs, GAA plus rumen-protected methionine improved water holding capacity, meat color a* and b* values; and decreased shear force and cooking loss [23]. However, inconsistency was reported in broilers where GAA did not affect L*, a*, b*, cooking loss, drip loss, shear force, and chemical composition in the breast muscle [20,22,24].

The changes in meat pH and textural characteristics in GAA groups of the present study may be related to the higher content of adenine nucleotide, including ATP, ADP, and AMP. As known, meat quality is greatly determined through biochemical changes occurring in the muscle during its conversion to meat; stored energy contributes greatly to the distinct contractile and metabolic properties of the skeletal muscle; and these are key to imparting a unique set of characteristics to meat appearance, ability to retain moisture, and texture [25,26]. Indeed, GAA lowered ultimate pH and the severity of wooden breast myopathy in broilers [27]. The increases in creatine, phosphocreatine, and ATP in GAA treatments were related to creatine transporter and the metabolites of amino acids, free amino acids, and energy in the pectoralis muscle of broilers [8,18]. Furthermore, GAA reduced muscle energy expenditure and delayed anaerobic glycolysis in transport-stressed broilers [20], which also partially explained the mechnism of GAA in the meat quality. In the present study, the creatine concentrations were not determined, which deserves further study.

## CONCLUSION

The addition of GAA at 0.04%, 0.08%, and 0.12% increased final body weight, carcass weight, the density and area of quadriceps femoris fiber, and meat color a* and b*, ATP, and TAN in longissimus and quadriceps. Among the three doses, T3 showed more significant effects on these parameters. It is concluded that GAA can improve the growth performance, carcass traits, and muscle adenine nucleotide of rabbits.

## Figures and Tables

**Table 1:** Compositions and nutrient levels of the basal diet (air-dry basis)

Items	Content (%)
Ingredient	
Corn	6.0
Soybean meal	8.0
Barley	7.0
Wheat bran	15.0
Corn germ meal	16.0
Alfalfa powder	30.0
Soybean straw	15.0
CaHPO_4_	1.5
NaCl	0.5
Premix^1)^	1.0
Nutrient level^2)^	
Digestible energy (MJ/kg)	10.30
Crude protein	16.01
Ether extract	2.83
Crude fiber	16.25
Crude ash	8.31
Ca	0.95
Total P	0.45
Lysine	0.60
Methionine	0.37
Methionine +cysteine	0.65

1) Provided the following per kg of the diet: Vit A 8,000 IU, Vit D_3_ 1,500 IU, Vit E 45 mg, Vit K_3_ 2.0 mg, Vit B_1_ 1.0 mg, Vit B_2_ 3.0 mg, Vit B_6_ 1.5 mg, nicotinic acid 30 mg, pantothenic acid 50 mg, folic acid 0.5 mg, choline chloride 100 mg, Fe 50 mg, Cu 10 mg, Zn 50 mg, Mn 10 mg, I 0.5 mg, Se 0.05 mg, Lysine 1.5 g, Methionine 0.5 g.

2) Digestible energy was calculated according to Chinese feedstuff database [14] and others were determined values.

**Table 2:** Effect of guanidinoacetic acid on the growth performance of meat-type rabbits

Item	Treatments^1)^				SEM	p-values		
	
	CON	T1	T2	T3		Post-Hoc	Linear	Quadratic
Guanidinoacetic acid (%)	0	0.04	0.08	0.12	-	-	-	-
Initial body weight (g)	936	941	933	937	6.982	0.876	0.326	0.438
Final body weight (kg)	2.47^c^	2.57^b^	2.60^ab^	2.64^a^	0.021	0.035	0.051	0.427
Daily weight gain (g/d)	38.5^b^	40.6^b^	42.1^ab^	43.0^a^	0.672	0.041	0.022	0.321
Daily feed intake (g/d)	165	175	179	179	6.643	0.053	0.526	0.569
Feed/gain	4.29^a^	4.30^a^	4.24^a^	4.18^b^	0.020	0.042	0.073	0.048
Survival rate (%)	91.7	93.3	93.3	91.7	12.68	0.527	0.603	0.711
Diarrhea (%)	11.0	7.0	9.0	8.0	7.324	0.703	0.324	0.054

SEM, standard error of the mean.

1) CON, control; T1, guanidinoacetic acid addition at 0.04% of diet; T2, guanidinoacetic acid addition at 0.08% of diet; T3, guanidinoacetic acid addition at 0.12% of diet.

a–c Means in a row with different superscripts are significantly different (p<0.05).

**Table 3:** Effect of guanidinoacetic acid on the carcass and myofiber traits of meat-type rabbits

Item	Treatment^1)^				SEM	p-values		
	
	CON	T1	T2	T3		Post-Hoc	Linear	Quadratic
Guanidinoacetic acid (%)	0	0.04	0.08	0.12	-	-	-	-
Carcass traits								
Pre-slaughter weight (kg)	2.47^c^	2.57^b^	2.60^ab^	2.64^a^	0.010	0.033	0.047	0.233
Hot carcass weight (kg)	1.58^c^	1.72^b^	1.74^ab^	1.76^a^	0.013	0.037	0.051	0.301
Commercial carcass (kg)	1.48^d^	1.54^c^	1.60^b^	1.67^a^	0.016	0.005	0.048	0.285
Dressing percentage (%)	59.8^b^	60.0^b^	61.7^ab^	63.1^a^	0.892	0.028	0.055	0.366
Reference carcass (kg)	1.15^d^	1.23^c^	1.27^b^	1.29^a^	0.009	0.031	0.059	0.627
Longissimus dorsi fiber								
Density (fiber/mm^2^)	514^c^	539^bc^	549^ab^	565^a^	10.02	0.043	0.038	0.552
Area (10^–3^ mm^2^/fiber)	1.74^c^	1.85^bc^	1.92^b^	2.04^a^	0.031	0.004	0.029	0.407
Quadriceps femoris fiber								
Density (fiber/mm^2^)	489^c^	524^b^	541^ab^	557^a^	10.27	0.008	0.065	0.508
Area (10–3 mm^2^/fiber)	1.77^d^	1.89^c^	1.98^b^	2.12^a^	0.029	0.002	0.023	0.436

SEM, standard error of the mean.

1) CON, control; T1, guanidinoacetic acid addition at 0.04% of diet; T2, guanidinoacetic acid addition at 0.08% of diet; T3, guanidinoacetic acid addition at 0.12% of diet.

a–d Means in a row with different superscripts are significantly different (p<0.05).

**Table 4:** Effect of guanidinoacetic acid on the meat quality of meat-type rabbits

Item		Treatment^1)^				SEM	p-value		
	
		CON	T1	T2	T3		Post-Hoc	Linear	Quadratic
Guanidinoacetic acid (%)		0	0.04	0.08	0.12	-	-	-	-
Longissimus dorsi									
Meat color	L*	43.6^c^	45.5^bc^	49.2^a^	48.8^a^	1.011	0.046	0.469	0.042
	a*	7.03^b^	7.29^a^	7.28^a^	7.46^a^	0.069	0.003	0.256	0.327
	b*	8.41	8.49	8.52	8.81	0.086	0.298	0.300	0.567
pH (24 h)									
Shear force (kg/cm^2^)									
Cooked meat rate (%)									
Quadriceps femoris									
Meat color	L*	58.3	59.3	58.8	59.2	1.602	0.219	0.560	0.209
	a*	8.14^c^	8.77^b^	8.83^b^	10.3^a^	0.043	0.003	0.035	0.112
	b*	9.36	9.73	9.45	9.61	0.106	0.412	0.228	0.077
pH (24 h)		5.74	5.75	5.73	5.32	0.007	0.558	0.442	0.528
Shear force (kg/cm^2^)		6.02	6.19	6.21	6.13	0.032	0.105	0.405	0.214
Cooked meat rate (%)		63.6	63.7	63.6	63.8	1.257	0.790	0.619	0.632

SEM, standard error of the mean.

1) CON, control; T1, guanidinoacetic acid addition at 0.04% of diet; T2, guanidinoacetic acid addition at 0.08% of diet; T3, guanidinoacetic acid addition at 0.12% of diet.

a–c Means in a row with different superscripts are significantly different (p<0.05).

**Table 5:** Effect of guanidinoacetic acid on the muscle adenine nucleotides of meat-type rabbits

Item	Treatment^1)^				SEM	p-value		
	
	CON	T1	T2	T3		Post-Hoc	Linear	Quadratic
Guanidinoacetic acid (%)	0	0.04	0.08	0.12	-	-	-	-
Longissimus dorsi								
Adenosine triphosphate (μ/g)	70.0^d^	97.8^c^	123^b^	158^a^	6.223	<0.001	0.018	0.322
Adenosine diphosphate (μ/g)	262^b^	279^ab^	285^a^	290^a^	7.181	0.007	0.025	0.498
Adenosine monophosphate (μ/g)	480	485	496	503	13.39	0.053	0.066	0.279
Total adenine nucleotide (μ/g)	812^d^	862^c^	904^b^	951^a^	12.98	<0.001	0.021	0.369
Adenylate energy charges	0.25^c^	0.28^b^	0.29^b^	0.32^a^	0.010	0.008	0.100	0.323
Quadriceps femoris								
Adenosine triphosphate (μ/g)	90.6^b^	103^b^	151^a^	159^a^	9.921	0.003	0.062	0.289
Adenosine diphosphate (μ/g)	273^b^	296^ab^	302^ab^	311^a^	10.54	0.011	0.058	0.317
Adenosine monophosphate (μ/g)	504^b^	521^ab^	536^ab^	565^a^	14.37	0.010	0.020	0.366
Total adenine nucleotide (μ/g)	868^c^	920^b^	989^a^	1,035^a^	17.36	0.001	0.002	0.324
Adenylate energy charges	0.26^b^	0.27^b^	0.31^a^	0.30^a^	0.012	0.012	0.119	0.042

SEM, standard error of the mean.

1) CON, control; T1, guanidinoacetic acid addition at 0.04% of diet; T2, guanidinoacetic acid addition at 0.08% of diet; T3, guanidinoacetic acid addition at 0.12% of diet.

a–d Means in a row with different superscripts are significantly different (p<0.05).

## References

[1] Ostojic SM (2016). Guanidinoacetic acid as a performance-enhancing agent. Amino Acids.

[2] Ostojic SM (2022). Safety of dietary guanidinoacetic acid: a villain of a good guy?. Nutrients.

[3] Ostojic SM (2022). Cataloguing guanidinoacetic acid content in various foods. Int J Vitam Nutr Res.

[4] Ahmed-Farid OA, Salah AS, Nassan MA, El-Tarabany MS (2021). Performance, carcass yield, muscle amino acid profile, and levels of brain neurotransmitters in aged laying hens fed diets supplemented with guanidinoacetic acid. Animals (Basel).

[5] Sharma NK, Cadogan DJ, Chrystal PV (2022). Guanidinoacetic acid as a partial replacement to arginine with or without betaine in broilers offered moderately low crude protein diets. Poult Sci.

[6] Speer HF, Grant MS, Miesner MD, Titgemeyer EC (2022). Effect of guanidinoacetic acid supplementation on nitrogen retention and methionine methyl group flux in growing steers fed corn-based diets. J Anim Sci.

[7] Wang L, Wang Y, Xu D, He L, Zhu X, Yin J (2022). Dietary guanidinoacetic acid supplementation improves water holding capacity and lowers free amino acid concentration of fresh meat in finishing pigs fed with various dietary protein levels. Anim Nutr.

[8] Zhang B, Liu N, Kang K (2022). Dietary guanidineacetic acid supplementation ameliorated meat quality and regulated muscle metabolism of broilers subjected to pre-slaughter transport stress by metabolomics analysis. Poult Sci.

[9] Lu Y, Zou T, Wang Z (2020). Dietary guanidinoacetic acid improves the growth performance and skeletal muscle development of finishing pigs through changing myogenic gene expression and myofibre characteristics. J Anim Physiol Anim Nutr (Berl).

[10] Zhu Z, Gu C, Hu S, Li B, Zeng X, Yin J (2020). Dietary guanidinoacetic acid supplementation improved carcass characteristics, meat quality and muscle fibre traits in growing–finishing gilts. J Anim Physiol Anim Nutr (Berl).

[11] Li F, Xie H, Liu L (2021). Nutrient requirement of meat-type rabbits. China Agric Ind Stand.

[12] Qin Y, Du Y, Gu X (2002). The technical specification for feeding and management of meat-type rabbits. China Agric Ind Stand.

[13] Wang J, Luo Y, Li P, Zhang F, Liu N (2021). Effect of Salvia miltiorrhiza aerial parts on growth performance, nutrient digestibility, and digestive enzymes in rabbits. Anim Biosci.

[14] Xiong B, Luo Q, Zheng S (2020). Compositions and nutritive values of feedstuffs in China. China Feed.

[15] Hou Y, Yao K, Wang L (2011). Effects of α-ketoglutarate on energy status in the intestinal mucosa of weaned piglets chronically challenged with lipopolysaccharide. Br J Nutr.

[16] Ostojic SM, Ratgeber L, Olah A, Betlehem J, Acs P (2020). Guanidinoacetic acid deficiency: a new entity in clinical medicine?. Int J Med Sci.

[17] Ostojic SM (2022). Guanidinoacetic acid as a nutritional adjuvant to multiple sclerosis therapy. Front Hum Neurosci.

[18] He DT, Gai XR, Yang LB (2018). Effects of guanidinoacetic acid on growth performance, creatine and energy metabolism, and carcass characteristics in growing-finishing pigs. J Anim Sci.

[19] Majdeddin M, Golian A, Kermanshahi H, De Smet S, Michiels J (2018). Guanidinoacetic acid supplementation in broiler chickens fed on corn-soybean diets affects performance in the finisher period and energy metabolites in breast muscle independent of diet nutrient density. Br Poult Sci.

[20] Zhang L, Li J, Wang X, Zhu XD, Gao F, Zhou GH (2019). Attenuating effects of guanidinoacetic acid on preslaughter transport-induced muscle energy expenditure and rapid glycolysis of broilers. Poult Sci.

[21] Zhao W, Li J, Xing T, Zhang L, Gao F (2021). Effects of guanidinoacetic acid and complex antioxidant supplementation on growth performance, meat quality, and antioxidant function of broiler chickens. J Sci Food Agric.

[22] Jayaraman B, La KV, La H (2018). Supplementation of guanidinoacetic acid to pig diets: effects on performance, carcass characteristics, and meat quality. J Anim Sci.

[23] Yan Z, Yan Z, Liu S, Yin Y, Yang T, Chen Q (2021). Regulative mechanism of guanidinoacetic acid on skeletal muscle development and its application prospects in animal husbandry: a review. Front Nutr.

[24] Zhang J, Li H, Zhang G (2022). Supplementation of guanidinoacetic acid and rumen-protected methionine increased growth performance and meat quality of Tan lambs. Anim Biosci.

[25] Matarneh SK, Silva SL, Gerrard DE (2021). New insights in muscle biology that alter meat quality. Annu Rev Anim Biosci.

[26] Córdova-Noboa HA, Oviedo-Rondón EO, Sarsour AH (2018). Performance, meat quality, and pectoral myopathies of broilers fed either corn or sorghum based diets supplemented with guanidinoacetic acid. Poult Sci.

[27] Li J, Hou Y, Yi D (2015). Effects of tributyrin on intestinal energy status, antioxidative capacity and immune response to lipopolysaccharide challenge in broilers. Asian-Australas J Anim Sci.

